# Clinical outcomes after relapse during rituximab-based maintenance in ANCA-associated vasculitis: A single-center retrospective cohort study

**DOI:** 10.1371/journal.pone.0357836

**Published:** 2026-09-15

**Authors:** Makoto Yamaguchi, Hirokazu Sugiyama, Hiroshi Kinashi, Keisuke Kamiya, Genri Tagami, Masayoshi Toda, Takayuki Katsuno, Takahiro Imaizumi, Shogo Banno, Yasuhiko Ito, Takuji Ishimoto

**Affiliations:** 1 Department of Nephrology and Rheumatology, Aichi Medical University, Nagakute, Aichi, Japan; 2 Department of Nephrology and Rheumatology, Aichi Medical University Medical Center, Okazaki, Aichi, Japan; 3 Data Coordinating Center, Department of Advanced Medicine, Nagoya University Hospital, Nagoya, Japan; Prime Hospital LLC, UNITED ARAB EMIRATES

## Abstract

Relapse during rituximab (RTX)-based maintenance therapy remains a clinical problem in ANCA-associated vasculitis (AAV). Mycophenolate mofetil (MMF) has been investigated as an alternative; however, evidence regarding its use after relapse during RTX-based maintenance is limited. We aimed to retrospectively describe clinical outcomes in patients with AAV who relapsed during RTX-based maintenance according to whether MMF was included in the initial post-relapse treatment strategy. We retrospectively analyzed 17 patients with AAV who experienced their first relapse during RTX-based maintenance after remission induction. Baseline was defined as the time of the first relapse. Post-relapse treatment included glucocorticoid escalation and/or RTX re-administration; patients were categorized according to whether MMF was additionally included in the initial treatment strategy (MMF-added group, n = 9; MMF-not-added group, n = 8). All outcomes were evaluated descriptively. In the MMF-added group, MMF was initiated at a median of 0.4 months after the first relapse (interquartile range [IQR], 0.3–0.4 months; range, 0.3–0.5 months). The median age was 76 years (IQR, 73–81 years); 16 patients (94.1%) were MPO-ANCA-positive. During a median follow-up of 28 months (IQR, 21–33 months), all patients achieved remission after post-relapse treatment intensification. No second relapse occurred in the MMF-added group, whereas three patients in the MMF-not-added group experienced a second relapse. Glucocorticoids were discontinued in all nine patients in the MMF-added group, whereas in the MMF-not-added group, all eight patients continued glucocorticoids while they were managed without MMF. Severe infections requiring hospitalization occurred in none of the patients in the MMF-added group and in three patients in the MMF-not-added group. Because treatment allocation was non-randomized and post-relapse treatment strategies differed with respect to RTX scheduling, concomitant therapies, and physician-directed glucocorticoid tapering, the independent effect of MMF could not be determined. Therefore, these descriptive and hypothesis-generating findings warrant confirmation in future prospective studies.

## Introduction

Anti-neutrophil cytoplasmic antibody (ANCA)–associated vasculitis (AAV), particularly microscopic polyangiitis (MPA) and granulomatosis with polyangiitis (GPA), is a necrotizing small- to medium-vessel vasculitis characterized by multisystem involvement. Timely initiation of immunosuppressive therapy is essential for improving outcomes.

Rituximab (RTX) has demonstrated efficacy for remission induction and maintenance in AAV and is recommended in major clinical guidelines [1–3].

However, despite the effectiveness of RTX, relapses may occur during RTX-based maintenance therapy, particularly during glucocorticoid (GC) tapering and long-term follow-up [2–5]. The optimal strategy for managing patients who experience relapse during RTX maintenance therapy remains unclear.

Mycophenolate mofetil (MMF) has been investigated as an alternative immunosuppressive agent for remission induction and maintenance in AAV, with some studies reporting clinical benefits in selected patients [6,7]. In addition, MMF has been investigated in combination with RTX in other autoimmune diseases, where potential additive immunosuppressive effects have been reported [8–10]. However, evidence regarding post-relapse treatment strategies incorporating MMF during RTX-based maintenance therapy in AAV remains limited.

Therefore, we retrospectively described clinical outcomes in patients with AAV who experienced a first relapse during RTX-based maintenance according to whether MMF was included in the initial post-relapse treatment strategy, focusing on second relapse, glucocorticoid withdrawal, and safety.

## Materials & methods

### Study design and patients

This single-center retrospective cohort study was conducted at the Department of Nephrology and Rheumatology, Aichi Medical University, Japan. Patients aged ≥ 20 years who were diagnosed with AAV, received RTX for remission induction and subsequent maintenance therapy between 2014 and 2022, and experienced their first relapse during RTX-based maintenance were eligible for the study. AAV was diagnosed according to the 2012 revised Chapel Hill Consensus Conference nomenclature, and patients were classified as having MPA or GPA using the European Medicines Agency vasculitis classification algorithm [11].

Patients were categorized according to the treatment strategy selected during the initial management of the first relapse. Patients for whom MMF was included in the initial post-relapse treatment strategy were classified into the MMF-added group, whereas those initially managed without MMF were classified into the MMF-not-added group. Patients in the MMF-not-added group who subsequently received MMF after a second relapse retained their original group assignment. No prespecified landmark time point was used for group assignment.

### Post-relapse treatment

Before the first relapse, RTX was administered for remission induction at one or two doses of 375 mg/m², followed by fixed-schedule maintenance every 6 months. For all post-relapse treatment, decisions were made by the treating physicians, and no standardized protocol was followed. Treatment intensification at the first relapse consisted of glucocorticoid escalation and/or RTX re-administration. When RTX was re-administered, a single dose of 375 mg/m² was used. Subsequent RTX maintenance was administered either on a fixed schedule of 375 mg/m² every 6 months or using a tailored strategy guided primarily by the reappearance of CD19-positive B cells; ANCA titer trends were used only as supportive information. MMF, when selected, was initiated at 1,000 mg/day in two divided doses. Intravenous methylprednisolone pulse therapy and avacopan were used at the discretion of the treating physicians. Trimethoprim–sulfamethoxazole prophylaxis against *Pneumocystis jirovecii* pneumonia was administered unless contraindicated.

### Measurements

Baseline characteristics were collected at the initiation of post-relapse treatment intensification for the first relapse. These included age, sex, AAV subtype, ANCA specificity, estimated glomerular filtration rate (eGFR, mL/min/1.73 m^2^; calculated as 194 × [serum creatinine]^−1.094^ × [age]^−0.287^ × 0.739 if female) [12], Birmingham Vasculitis Activity Score version 3 (BVAS v3) [13], relapse phenotype, organ involvement, serum immunoglobulin G (IgG) levels, prior cumulative RTX dose, prednisolone dose at relapse, and concomitant medications.

Relapse was defined as new or recurrent disease activity attributable to AAV that required intensification of immunosuppressive therapy after alternative explanations, including infection, drug toxicity, chronic organ damage, and hemodynamic kidney dysfunction, had been excluded [14]. Severe relapse was defined as the occurrence of a new or recurrent major BVAS/WG item or other organ- or life-threatening disease activity [15]. Relapses requiring treatment intensification that did not meet these criteria were classified as non-severe. Renal, pulmonary, and neurological relapses were determined on the basis of new or worsening organ-specific findings attributable to active vasculitis. ANCA positivity or an increase in ANCA titer alone was not considered sufficient to define relapse or prompt treatment intensification.

Serum IgG levels and MPO-ANCA and PR3-ANCA titers were recorded at diagnosis, at the first relapse, and at 3, 6, 12, and 24 months after post-relapse treatment intensification. ANCA titers were measured using a direct antigen-specific enzyme-linked immunosorbent assay. Post-relapse treatment and monitoring data included RTX dose and scheduling strategy, CD19-positive B-cell monitoring, MMF initiation and dose, avacopan exposure, intravenous methylprednisolone pulse therapy, and oral glucocorticoid use. Glucocorticoid doses at relapse and at 3, 6, and 12 months after relapse, glucocorticoid withdrawal, and time to withdrawal were also recorded.

### Outcomes

The main descriptive outcome was a second relapse after the first relapse. Other outcomes included clinical remission after post-relapse treatment intensification, glucocorticoid withdrawal and time to withdrawal, severe infection requiring hospitalization, hypogammaglobulinemia, all-cause mortality, initiation of kidney replacement therapy, and bone fracture. Clinical remission was defined as a BVAS v3 score of 0 with a daily prednisone-equivalent dose of ≤ 7.5 mg, and hypogammaglobulinemia was defined as a serum IgG level of < 500 mg/dL.

Treatment-emergent adverse events were defined as new or worsening adverse events occurring after post-relapse treatment intensification. Severe infection was defined as an infection requiring hospitalization and intravenous antimicrobial therapy. Adverse events were attributed to a specific treatment only when the timing, clinical course, treatment discontinuation, or dose reduction supported a plausible relationship. All patients were followed until June 1, 2025, death, or the last available hospital visit, whichever occurred first.

### Statistical analysis

Continuous variables were summarized as medians and interquartile ranges (IQRs), and categorical variables were summarized as numbers and percentages. No formal between-group hypothesis testing, multivariable adjustment, propensity-score analysis, or time-to-event modeling was performed because of the small sample size, few outcome events, non-randomized treatment allocation, and heterogeneity in post-relapse treatment strategies. All analyses were descriptive and hypothesis-generating. No sample size or power calculation was prespecified; the sample size was determined by the number of eligible patients during the study period. Statistical analyses were performed using JMP version 14.0.0 (SAS Institute, Cary, NC, USA) and Stata version 13.0 (StataCorp, College Station, TX, USA).

### Ethics approval

The study protocol was approved by the Ethics Committee of Aichi Medical University (approval number 2018-H183, August 10, 2018) and was conducted in accordance with the Declaration of Helsinki.

### Patient consent

The requirement for written informed consent was waived because of the retrospective nature of the study. Study information was disclosed through the institutional opt-out process, and patients were provided with an opportunity to decline participation.

### Reporting guidelines

The manuscript was prepared following the STROBE reporting guidelines. A completed STROBE cohort checklist has been uploaded.

## Results

### Patient flow and baseline characteristics at first relapse

**Fig 1:**
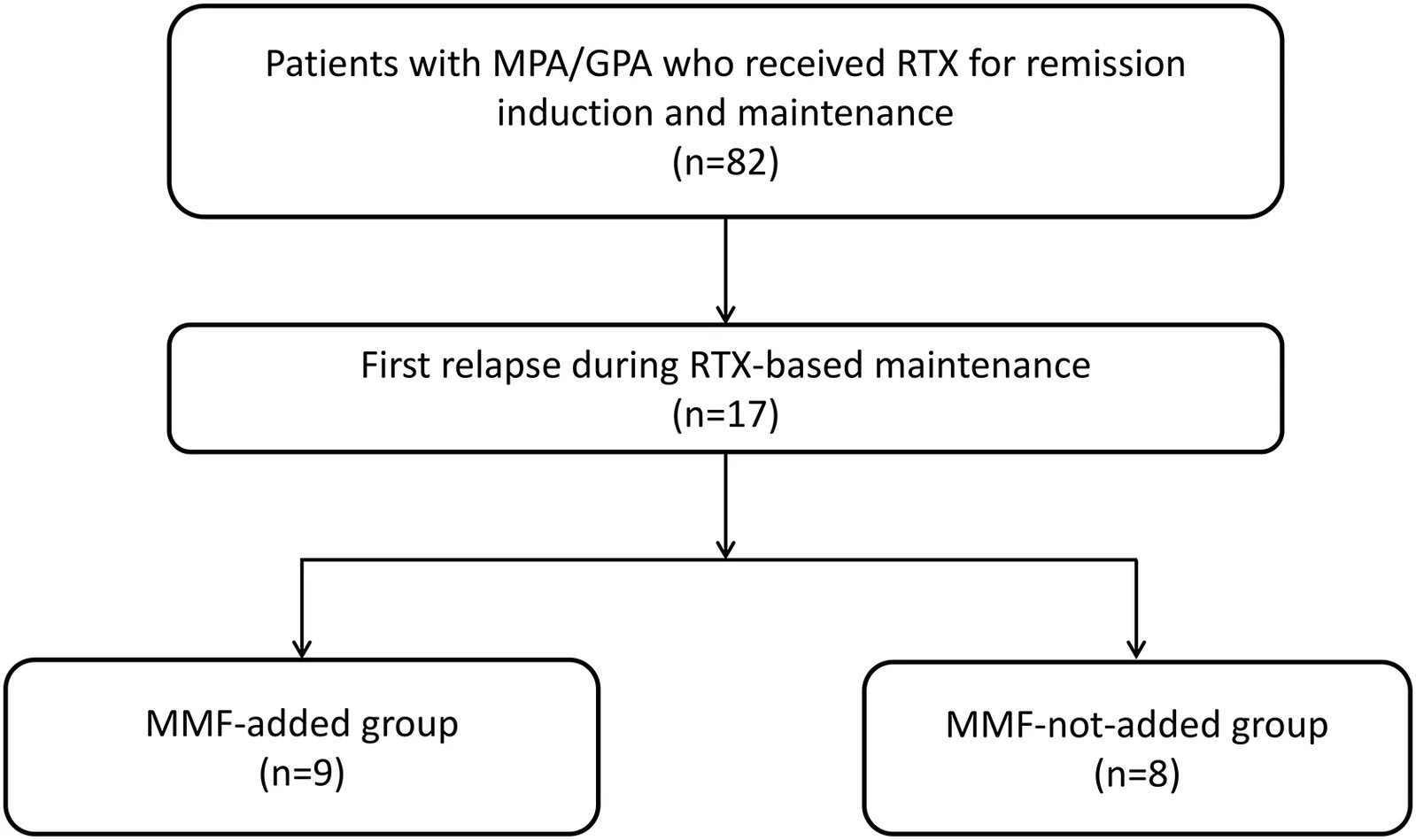
Patient flow diagram. Of the total 82 patients with MPA/GPA who received RTX for remission induction and maintenance, 17 experienced a first relapse during RTX-based maintenance and were included in the present analysis. After the first relapse, nine and eight patients were classified into the MMF-added group and MMF-not-added group, respectively, according to the initial post-relapse treatment strategy.

Patient flow and baseline characteristics at the first relapse are summarized in Fig 1 and Table 1. Among 82 patients with AAV who received RTX for remission induction and subsequent maintenance therapy, 17 experienced a first relapse during RTX-based maintenance and were included in the present analysis. Nine and eight patients were categorized into the MMF-added group and MMF-not-added group, respectively, according to the initial post-relapse treatment strategy.

**Table 1:** Clinical characteristics at first relapse.

	MMF-added group (n = 9)	MMF-not-added group (n = 8)
Clinical characteristics		
Age (years)	80 (75–81)	75 (72–76)
Male sex	6 (66.7)	5 (62.5)
Disease duration (months)	18 (12–27)	25 (16–35)
eGFR (mL/min/1.73 m^2^)	26.9 (19.8–33.0)	30.1 (18.2–35.1)
On maintenance dialysis at first relapse	0 (0)	1 (12.5)
AAV subtype		
MPA	8 (88.9)	8 (100)
GPA	1 (11.1)	0 (0)
Antibody		
MPO-ANCA	8 (88.9)	8 (100)
PR3-ANCA	1 (11.1)	0 (0)
BVAS v3	14 (11–17)	13 (10–16)
Relapse type		
Severe	7 (77.8)	5 (62.5)
Non-severe	2 (22.2)	3 (37.5)
Organ involvement		
General	9 (100)	8 (100)
Ear, nose, and throat	0 (0)	1 (12.5)
Lung	2 (22.2)	1 (12.5)
Kidney	4 (44.4)	3 (37.5)
Nervous system	1 (11.1)	3 (37.5)
Prior RTX cumulative dose (g)	1.5 (1.0–2.0)	1.5 (1.5–1.9)
Prednisolone dose (mg/day) at relapse	2.5 (2.3–3.5)	2.5 (1.3–3.8)

Continuous data are presented as medians (interquartile ranges), whereas categorical data are expressed as numbers (percentages). AAV, ANCA-associated vasculitis; MPA, microscopic polyangiitis; GPA, granulomatosis with polyangiitis; MMF, mycophenolate mofetil; RTX, rituximab; eGFR, estimated glomerular filtration rate; MPO, myeloperoxidase; PR3, proteinase 3; ANCA, anti-neutrophil cytoplasmic antibody; BVAS, Birmingham Vasculitis Activity Score.

Of the 17 patients, 16 (94.1%) were classified as having MPA, and 1 (5.9%) was classified as having GPA. The patient with GPA was PR3-ANCA-positive and was included in the MMF-added group. The median age was 76 years (IQR, 73–81 years), and 11 patients (64.7%) were male. Sixteen patients (94.1%) were MPO-ANCA-positive, and 1 (5.9%) was PR3-ANCA-positive. The median eGFR at the first relapse was 29 mL/min/1.73 m² (IQR, 19–34), and the median BVAS v3 score was 14 (IQR, 11–16). At the first relapse, organ involvement included kidneys in seven patients (41.2%), lungs in three patients (17.6%), and nervous system in four patients (23.5%). One patient was receiving maintenance dialysis.

All patients showed either reappearance or elevation of serum ANCA titers at the first relapse; however, relapse was determined on the basis of clinical disease activity requiring treatment intensification and not on ANCA titers alone. At the first relapse, 15 patients were receiving glucocorticoids at a median prednisolone-equivalent dose of 2.5 mg/day (IQR, 2.0–3.5 mg/day), whereas two patients had discontinued glucocorticoids.

### Immunosuppressive treatment after first relapse

**Fig 2:**
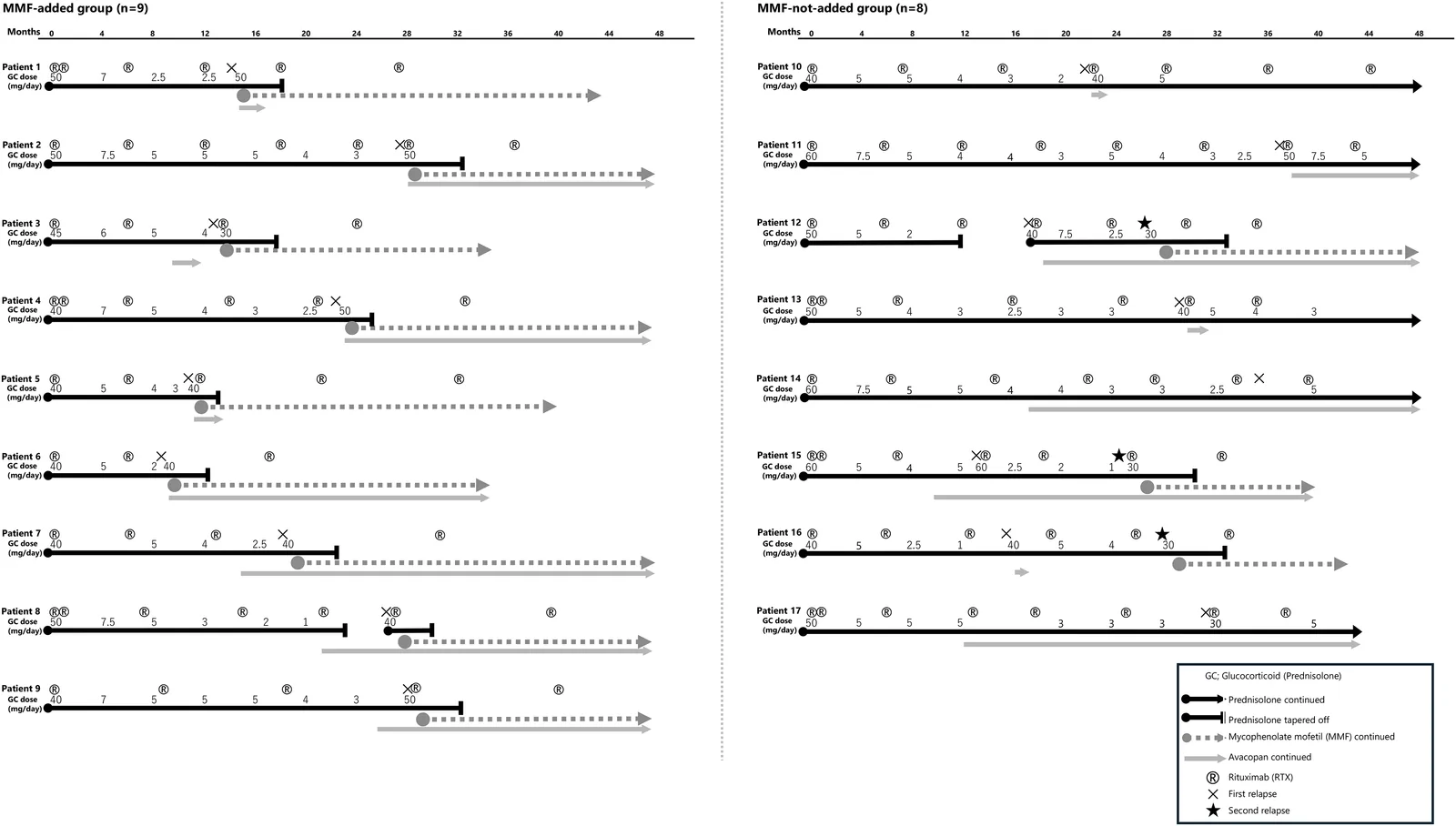
Timeline of individual immunosuppressive treatment courses from diagnosis through follow-up. The timeline shows RTX administration and dosing strategy, MMF exposure, avacopan exposure, prednisolone dose and tapering, glucocorticoid withdrawal, and relapse events in patients categorized according to the initial post-relapse treatment strategy. Gray arrows indicate avacopan treatment, and short gray arrows indicate early discontinuation of avacopan. The × symbol indicates the first relapse used for group allocation, and the ★ symbol indicates a second relapse during follow-up. In the MMF-not-added group, Patients 12, 15, and 16 experienced a second relapse and subsequently received MMF.

The individual treatment courses are illustrated in Fig 2, and post-relapse treatments are summarized in Table 2. After the first relapse, treatment intensification included glucocorticoid escalation and/or RTX re-administration, with MMF added as part of the initial post-relapse treatment strategy in nine patients. RTX was re-administered after the first relapse in 11 patients: 5 of 9 patients in the MMF-added group and 6 of 8 patients in the MMF-not-added group. All 11 patients received a single RTX dose of 375 mg/m²; none received the standard full re-induction regimen.

**Table 2:** Post-relapse treatments.

	MMF-added group (n = 9)	MMF-not-added group (n = 8)
Treatment after first relapse		
RTX re-administration after first relapse	5 (55.6)	6 (75.0)
Fixed-schedule RTX after first relapse	0	8 (100)
Tailored RTX after first relapse	9 (100)	0
Time from relapse to MMF initiation, months	0.4 (0.3–0.4), range 0.3–0.5	
Avacopan use before the first relapse	4 (44.4)	3 (37.5)
Avacopan newly initiated after the first relapse	5 (55.6)	5 (62.5)
Time from first relapse to avacopan initiation, months	0.4 (0.3–0.6)	0.5 (0.4–0.7)
Adverse events attributed to avacopan	3 (33.3)	3 (37.5)
Hepatotoxicity	3 (33.3)	1 (12.5)
Diarrhea	0 (0)	2 (25.0)
Avacopan discontinuation due to adverse events	3 (33.3)	3 (37.5)
Time to avacopan discontinuation (months)	1.2 (1.1–1.8)	0.5 (0.3–0.9)
mPSL pulse therapy	1 (11.1)	0 (0)
Prednisolone dose (mg/day) during follow-up period after first relapse		
Initial dose	40 (40–50)	40 (40–40)
at 3 months	1 (0–2.3)	5 (5.0–5.8)
at 6 months	0	5 (4.0–5.0)
at 12 months	0	4 (3.0–5.0)
Off prednisolone	9 (100)	0 (0)
Time to prednisolone withdrawal from relapse (months)	3.8 (2.5–4.3)	

Continuous data are presented as medians (interquartile ranges), whereas categorical data are expressed as numbers (percentages). When RTX was administered after the first relapse, it was given as a single dose of 375 mg/m². The MMF-added group included patients in whom MMF was added as part of the initial post-relapse treatment strategy. The MMF-not-added group included patients in whom MMF was not added during the initial post-relapse treatment period; patients who subsequently received MMF after a second relapse were analyzed according to the initial strategy. MMF, mycophenolate mofetil; RTX, rituximab; mPSL, methylprednisolone; PSL, prednisolone.

In the MMF-added group, MMF was initiated at a median of 0.4 months after the first relapse (IQR, 0.3–0.4 months; range, 0.3–0.5 months). No second relapse, severe infection requiring hospitalization, or death occurred between the first relapse and MMF initiation in the MMF-added group. All nine patients in this group switched from fixed-schedule RTX maintenance every 6 months to a tailored RTX re-administration strategy primarily guided by the reappearance of CD19-positive B cells, with dosing intervals extended beyond 6 months. In contrast, all eight patients in the MMF-not-added group continued fixed-schedule RTX maintenance every 6 months.

The median initial prednisolone dose after the first relapse was 40 mg/day in both groups (IQR, 40–50 mg/day in the MMF-added group and 40–40 mg/day in the MMF-not-added group). At 3, 6, and 12 months after the first relapse, the median prednisolone doses were 1, 0, and 0 mg/day, respectively, in the MMF-added group and 5, 5, and 4 mg/day, respectively, in the MMF-not-added group. Glucocorticoid tapering was not standardized and was determined by the treating physicians.

Avacopan had been administered before the first relapse in four patients in the MMF-added group and three patients in the MMF-not-added group. It was newly initiated after the first relapse in five patients in each group. Avacopan-associated adverse events and treatment discontinuations are reported in the Safety section.

### Clinical remission and second relapse following the first relapse

The clinical outcomes after the first relapse are summarized in Table 3. During a median follow-up of 28 months (IQR, 21–33 months), all 17 patients achieved clinical remission after post-relapse treatment intensification. No second relapse occurred in the MMF-added group, whereas three of eight patients in the MMF-not-added group experienced a second relapse.

**Table 3:** Outcomes after first relapse.

	MMF-added group (n = 9)	MMF-not-added group (n = 8)
Outcomes		
Clinical remission	9 (100)	8 (100)
Second relapse	0 (0)	3 (37.5)
Death	0 (0)	1 (12.5)
ESRD (newly initiated dialysis)	0 (0)	0 (0)
Hospitalization due to severe infection	0 (0)	3 (37.5)
Bone fracture	0 (0)	2 (25.0)
Observation period from first relapse (months)	28 (23–30)	30 (11–36)

Continuous data are presented as medians (interquartile ranges), and categorical data are presented as numbers (percentages). Clinical remission was defined as a BVAS v3 score of 0 with a daily prednisone-equivalent dose of ≤ 7.5 mg. Severe infection was defined as an infection requiring hospitalization and intravenous antimicrobial therapy. MMF, mycophenolate mofetil; BVAS v3, Birmingham Vasculitis Activity Score version 3.

The three patients who experienced a second relapse subsequently received MMF as part of their treatment strategy. Glucocorticoids were later discontinued in all three patients, with no further documented relapse during the remaining follow-up period.

### Glucocorticoid discontinuation

Glucocorticoids were discontinued in all nine patients in the MMF-added group at a median of 3.8 months after the first relapse (IQR, 2.5–4.3 months). In the MMF-not-added group, none of the eight patients discontinued glucocorticoids while they were managed without MMF. The three patients who subsequently received MMF after a second relapse later discontinued glucocorticoids, with no further documented relapse during the remaining follow-up period.

### Other outcomes after first relapse

Severe infection requiring hospitalization was not observed in any patient in the MMF-added group, whereas it occurred in three patients in the MMF-not-added group. No deaths occurred in the MMF-added group, whereas one patient in the MMF-not-added group died during follow-up. Bone fractures occurred in none of the patients in the MMF-added group and in two patients in the MMF-not-added group. No patient in either group newly required kidney replacement therapy.

ANCA seroconversion to negativity was observed in seven of nine patients in the MMF-added group and two of eight patients in the MMF-not-added group (S1 Table). Because ANCA titers have limited value for predicting relapse in individual patients, these findings were interpreted as supportive serological observations rather than as evidence of treatment efficacy.

### Safety associated with immunosuppressive treatment

Adverse events attributed to avacopan occurred in three patients in both the MMF-added group and MMF-not-added group. In the MMF-added group, all three events were hepatotoxicity. Conversely, in the MMF-not-added group, hepatotoxicity occurred in one patient, and diarrhea occurred in 2 patients. Avacopan was discontinued because of these adverse events in all six patients. The median time from avacopan initiation to discontinuation was 1.2 months (IQR, 1.1–1.8 months) in the MMF-added group and 0.5 months (IQR, 0.3–0.9 months) in the MMF-not-added group.

No adverse events were clearly attributed to MMF in the MMF-added group. Hypogammaglobulinemia developed in no patients in the MMF-added group and in two patients in the MMF-not-added group (S2 Table).

## Discussion

This study was a small, single-center retrospective cohort study of patients with AAV who experienced relapse during RTX-based maintenance therapy. Sustained remission and glucocorticoid discontinuation were observed in selected patients managed with an individualized post-relapse treatment strategy incorporating MMF. However, treatment allocation was non-randomized, and post-relapse treatment strategies differed with respect to RTX administration, concomitant therapies, and physician-directed glucocorticoid tapering. Therefore, the findings of this study should be interpreted as descriptive and hypothesis-generating.

With regard to RTX, re-administration after the first relapse consisted exclusively of a single dose of 375 mg/m² rather than a conventional re-induction regimen. This lower-intensity approach reflected physician judgment based on factors such as age, kidney function, previous RTX exposure, immunoglobulin levels, and concerns regarding infection and treatment-related toxicity. However, this dosing strategy may have influenced subsequent outcomes, and because no patients received a conventional RTX re-induction regimen [1–3], its contribution could not be separated from that of the other components of the post-relapse treatment strategy. Cyclophosphamide was also generally avoided because of concerns regarding infection and treatment-related toxicity.

MMF has been investigated as an alternative immunosuppressive agent for remission induction and maintenance in AAV [6,7]. In addition, combination therapy with MMF and RTX has been investigated in other autoimmune diseases [8–10]. One theoretical rationale for incorporating MMF into a post-relapse treatment strategy during RTX-based maintenance therapy is that MMF suppresses lymphocyte proliferation and B-cell differentiation through mechanisms distinct from RTX-mediated B-cell depletion [16,17].

In the present study, glucocorticoid discontinuation was achieved in all patients in the MMF-added group. In addition, all patients in this group were switched from fixed-schedule RTX maintenance every 6 months to an individualized strategy in which RTX dosing intervals were extended beyond 6 months. An individualized treatment strategy incorporating MMF may have been associated with reduced glucocorticoid exposure and cumulative RTX exposure during follow-up.

However, neither glucocorticoid tapering nor extension of RTX dosing intervals was standardized, and both were determined by the treating physicians. In addition, avacopan was used in all patients in both groups; however, the primary publication of the ADVOCATE trial was retracted in 2026, and regulatory authorities have also raised concerns regarding the reliability of the evidence supporting its efficacy [18–20]. Therefore, the extent to which avacopan contributed to glucocorticoid discontinuation in the present study remains unclear. Taken together, the observed outcomes cannot be attributed to the independent effect of MMF.

The MMF dose used in this cohort was 1,000 mg/day, which was lower than the 2–3 g/day evaluated in previous studies of AAV [6,7]. This dose was selected by the treating physicians based on considerations such as age and the risk of infection during concomitant RTX therapy. However, the optimal dose of MMF in patients with AAV who relapse during RTX-based maintenance therapy remains unknown and warrants further investigation.

The interpretation of ANCA titers also requires caution. All patients showed either reappearance of ANCA or an increase in ANCA titers at the first relapse. Following treatment intensification after the first relapse, ANCA seroconversion to negativity was observed in seven of nine patients (77.8%) in the MMF-added group, compared with two of eight patients (25.0%) in the MMF-not-added group. Because the utility of ANCA titers for predicting relapse in individual patients is limited, whether ANCA seroconversion to negativity was associated with subsequent maintenance of remission could not be determined from the present study. Therefore, these findings should be interpreted as supportive serological observations rather than as evidence of treatment efficacy [21].

Regarding safety, no severe infections, bone fractures, or hypogammaglobulinemia were observed in the MMF-added group. Although reduced glucocorticoid exposure and cumulative RTX exposure resulting from extended RTX dosing intervals may have contributed to these findings, the small sample size and differences in comorbidities and concomitant therapies between the groups preclude conclusions regarding the comparative safety of the treatment strategies. In addition, liver injury considered to be related to avacopan occurred in 4 of 17 patients (23.5%) in this cohort, consistent with the safety concern regarding postmarketing avacopan-associated liver injury identified by the FDA [22].

This study has some limitations. First, this was a single-center retrospective study that included only 17 patients, with few outcome events, which precluded adjusted analyses accounting for potential confounders. Second, treatment selection was based on physician judgment, and RTX administration, concomitant therapies, and glucocorticoid tapering strategies differed between the groups. Therefore, confounding by indication and selection bias could not be excluded, and the independent effect of MMF could not be assessed. Third, MMF was initiated 0.3–0.5 months after the first relapse, and no prespecified landmark time point was used for group assignment. Although no second relapse, severe infection requiring hospitalization, or death occurred before MMF initiation in the MMF-added group, MMF was initiated after the first relapse, and the possibility of immortal time bias could not be completely excluded. Finally, the cohort predominantly consisted of MPO-ANCA-positive patients and had a median age of 76 years, limiting the generalizability of these findings to younger patients and those with PR3-ANCA-positive GPA.

## Conclusion

In patients with AAV who relapsed during RTX-based maintenance therapy, sustained remission and glucocorticoid discontinuation were observed under an individualized post-relapse treatment strategy incorporating MMF. These findings are descriptive and hypothesis-generating and require confirmation in future prospective studies.

## Supporting information

S1 Table Serum ANCA titer during the observation period. (DOCX)

S2 Table Serum IgG levels during the observation period. (DOCX)

S1 Text De-identified individual-level clinical dataset underlying the main findings of this study. (XLSX)
