# Supplementary material for: Clinical outcomes after relapse during rituximab-based maintenance in ANCA-associated vasculitis: A single-center retrospective cohort study
Source: PLoS One. 2026 Sep 15;21(9):e0357836. doi: 10.1371/journal.pone.0357836 (PMC13577381; doi:10.1371/journal.pone.0357836)
Supplement: Supplementary file 1 [file pone.0357836.s001.docx]

S1 Table. Serum ANCA titer during the observation period

|  |  | ANCA titer (IU/mL) | | | | | | |  |
| --- | --- | --- | --- | --- | --- | --- | --- | --- | --- |
| Patients | Treatment group after first relapse | ANCA titer at Dx | ANCA titer at remission | ANCA titer at relapse | ANCA titer (3 months after relapse) | ANCA titer (6 months after relapse) | ANCA titer (12 months after relapse) | ANCA titer (24 months after relapse) |  |
|  |  |  |  |  |  |  |  |  |  |
| Case 1 | MMF-added | 162.4 | 11.2 | 72.6 | 18.7 | 0 | 0 | 0 |  |
| Case 2 | MMF-added | 78.4 | 20.8 | 46.8 | 8.2 | 3.8 | 0 | 0 |  |
| Case 3 | MMF-added | 94.5 | 7.5 | 22.4 | 23.9 | 7.2 | 0 | 0 |  |
| Case 4 | MMF-added | 69.8 | 14.8 | 13.3 | 0 | 0 | 0 | 0 |  |
| Case 5 | MMF-added | 104.6 | 8.8 | 27.6 | 7.4 | 0 | 0 | 0 |  |
| Case 6 | MMF-added | 115.3 | 18.8 | 48.8 | 14.3 | 8.9 | 4.6 | 5.1 |  |
| Case 7 | MMF-added | 79.4 | 27.2 | 22.5 | 0 | 0 | 0 | 0 |  |
| Case 8 | MMF-added | 64.2 | 4.6 | 6.8 | 0 | 0 | 0 | 0 |  |
| Case 9 | MMF-added | 136.5 | 12.4 | 33.8 | 24.8 | 5.2 | 4.8 | N/A |  |
| Case 10 | MMF-not-added | 316.7 | 58.3 | 110.4 | 86.5 | 71.2 | 48.8 | 21.6 |  |
| Case 11 | MMF-not-added | 44.3 | 10.8 | 30.2 | 18.3 | 13.8 | 21.1 | 14.6 |  |
| Case 12 | MMF-not-added | 63.6 | 4.1 | 12.8 | 11.4 | 9.6 | 10.4 | 8.9 |  |
| Case 13 | MMF-not-added | 50.4 | 0 | 10.2 | 32.2 | 18.2 | 19.7 | 31.2 |  |
| Case 14 | MMF-not-added | 85.8 | 9.2 | 11.3 | 9.6 | 4.8 | 6.2 | 9.3 |  |
| Case 15 | MMF-not-added | 108.4 | 10.6 | 43.9 | 16.5 | 8.8 | 11.4 | 13.2 |  |
| Case 16 | MMF-not-added | 74.2 | 4.8 | 25.3 | 10.8 | 12.7 | 16.1 | 11.4 |  |
| Case 17 | MMF-not-added | 89.1 | 9.9 | 10.2 | 8.9 | 3.6 | 5.9 | 8.4 |  |

MMF, mycophenolate mofetil; RTX, rituximab; MPO, myeloperoxidase; PR3, proteinase-3; ANCA, anti-neutrophil cytoplasmic antibody; N/A, not available

Case 4 presents the PR3-ANCA titer, while all other cases present MPO-ANCA titers.
