# Supplementary material for: Clinical outcomes after relapse during rituximab-based maintenance in ANCA-associated vasculitis: A single-center retrospective cohort study
Source: PLoS One. 2026 Sep 15;21(9):e0357836. doi: 10.1371/journal.pone.0357836 (PMC13577381; doi:10.1371/journal.pone.0357836)
Supplement: Supplementary file 2 [file pone.0357836.s002.docx]

S2 Table. Serum IgG levels during the observation period

|  |  | Serum IgG levels (mg/dL) | | | | | |  |
| --- | --- | --- | --- | --- | --- | --- | --- | --- |
| Patients | Treatment group after first relapse | Serum IgG levels at Dx | Serum IgG levels at first relapse | Serum IgG levels (3 months after first relapse) | Serum IgG levels (6 months after first relapse) | Serum IgG levels (12 months after first relapse) | Serum IgG levels (24 months after first relapse) |  |
|  |  |  |  |  |  |  |  |  |
| Case 1 | MMF-added | 1,748 | 718 | 668 | 749 | 854 | 802 |  |
| Case 2 | MMF-added | 1,530 | 1,104 | 988 | 1,058 | 1,130 | 1,039 |  |
| Case 3 | MMF-added | 1,884 | 1,430 | 1,446 | 1,438 | 1,360 | 1,429 |  |
| Case 4 | MMF-added | 998 | 604 | 694 | 788 | 833 | 997 |  |
| Case 5 | MMF-added | 1,329 | 986 | 962 | 1,035 | 1,104 | 1,204 |  |
| Case 6 | MMF-added | 1,628 | 1,528 | 1,650 | 1,582 | 1,453 | 1,385 |  |
| Case 7 | MMF-added | 2,033 | 1,648 | 1,430 | 1,528 | 1,448 | 1,302 |  |
| Case 8 | MMF-added | 1,295 | 788 | 748 | 842 | 894 | 954 |  |
| Case 9 | MMF-added | 1,194 | 659 | 776 | 898 | 910 | N/A |  |
| Case 10 | MMF-not-added | 2,086 | 1,065 | 892 | 749 | 772 | 493 |  |
| Case 11 | MMF-not-added | 1,666 | 689 | 534 | 493 | 486 | 462 |  |
| Case 12 | MMF-not-added | 1,430 | 884 | 733 | 503 | 518 | N/A |  |
| Case 13 | MMF-not-added | 1,206 | 663 | 443 | 610 | 495 | 501 |  |
| Case 14 | MMF-not-added | 1,548 | 1,032 | 974 | 747 | 805 | 587 |  |
| Case 15 | MMF-not-added | 1,308 | 382 | 511 | 462 | 554 | 459 |  |
| Case 16 | MMF-not-added | 1,132 | 869 | 698 | 616 | 552 | 454 |  |
| Case 17 | MMF-not-added | 1,639 | 994 | 860 | 719 | 824 | 728 |  |

MMF, mycophenolate mofetil; RTX, rituximab; IgG, immunoglobulin G; N/A, not available
